# IgA natural antibodies are produced following T-cell independent B-cell activation following stroke

**DOI:** 10.1016/j.bbi.2020.09.014

**Published:** 2020-09-19

**Authors:** Jacob C. Zbesko, Jennifer Beischel Frye, Danielle A. Becktel, Diana K. Gerardo, Jessica Stokes, Kylie Calderon, Thuy-Vi V. Nguyen, Deepta Bhattacharya, Kristian P. Doyle

**Affiliations:** a Department of Immunobiology, University of Arizona, Tucson, AZ 85719, USA; b Department of Pediatrics, University of Arizona, Tucson, AZ 85719, USA; c Department of Neurology, University of Arizona, Tucson, AZ 85719, USA; d Arizona Center on Aging, University of Arizona, Tucson, AZ 85719, USA

**Keywords:** Stroke, IgA, Chronic inflammation, Thymus-independent type 2 antigen, Cognitive decline, B-lymphocytes, CD4+ helper T-lymphocytes

## Abstract

Up to 30% of stroke patients experience cognitive decline within one year of their stroke. There are currently no FDA-approved drugs that can prevent post-stroke cognitive decline, in part due to a poor understanding of the mechanisms involved. We have previously demonstrated that a B-lymphocyte response to stroke, marked by IgA + cells, can cause delayed cognitive dysfunction in mice and that a similar adaptive immune response occurs in the brains of some human stroke patients that suffer from vascular dementia. The stimuli which trigger B-lymphocyte activation following stroke, and their target antigens, are still unknown. Therefore, to learn more about the mechanisms by which B-lymphocytes become activated following stroke we first characterized the temporal kinetics of the B-lymphocyte, T-lymphocyte, and plasma cell (PC) response to stroke in the brain by immunohistochemistry (IHC). We discovered that B-lymphocyte, T-lymphocyte, and plasma cell infiltration within the infarct progressively increases between 2 and 7 weeks after stroke. We then compared the B-lymphocyte response to stroke in WT, MHCII^−/−^, CD4^−/−^, and MyD88^−/−^ mice to determine if B-lymphocytes mature into IgA + PCs through a T-lymphocyte and MyD88 dependent mechanism. Our data from a combination of IHC and flow cytometry indicate that following stroke, a population of IgA + PCs develops independently of CD4 + helper T-lymphocytes and MyD88 signaling. Subsequent sequencing of immunoglobulin genes of individual IgA + PCs present within the infarct identified a novel population of natural antibodies with few somatic mutations in complementarity-determining regions. These findings indicate that a population of IgA + PCs develops in the infarct following stroke by B-lymphocytes interacting with one or more thymus independent type 2 (TI-2) antigens, and that they produce IgA natural antibodies.

## 1. Introduction

Every year, nearly 800,000 people suffer an ischemic stroke in the United States alone (Waldman et al., 2020). Of these patients, approximately one third develop a delayed and progressive form of cognitive decline (Prencipe et al., 1997). Recently, using an experimental model of stroke, we demonstrated that B-lymphocytes play a role in the development of a similar cognitive decline in mice (Doyle et al., 2015). Ortega et al. have concomitantly discovered that stroke induces a rapid and sustained B-lymphocyte response to both neuronal and myelin peptide antigens (Ortega et al., 2015). However, the mechanisms by which B-lymphocytes are activated following stroke and the full repertoire of antigens that cause their maturation into plasma cells (PCs) are still not fully defined. To further our understanding of the role of B-lymphocytes following stroke, our goal in this study was to examine the mechanisms by which B-lymphocytes become activated, focusing specifically on the IgA component of the B-lymphocyte response to stroke.

We have previously shown that IgA + PCs are present within the infarct at 7 weeks following experimental stroke in mice (Doyle et al., 2015). IgA is an antibody isotype produced and secreted in large amounts in the intestinal mucosa, where it aids in the homeostasis of the microbiome (Donaldson et al., 2018; Mishima et al., 2019). Within the intestinal mucosa, IgA + PCs develop by both T-lymphocyte dependent and T-lymphocyte independent mechanisms (Bunker et al., 2017). Antibodies formed through the T-lymphocyte independent mechanism, called natural antibodies, have weaker binding capabilities, due to the lack of affinity maturation, than antibodies formed through the T-lymphocyte dependent mechanism (Palma et al., 2018). However, individual natural IgA antibodies are often polyreactive, endowed with the ability to bind to a wide array of antigens with diverse structures, including DNA, oxidized phospholipids, insulin, cardiolipin, lipopolysaccharide (LPS), and flagellin (Bunker et al., 2017; Quan et al., 1997; Wijburg et al., 2006).

Furthermore, IgA can be produced as a monomeric as well as a dimeric form, with the dimeric form the most prevalent (Snoeck et al., 2006). This means that despite their low affinity, polyreactive IgA natural antibodies are strongly polyvalent. This gives them the ability to agglutinate their targets, thereby enhancing phagocytic clearance, and, in the case of bacteria, preventing adherence to host cells (Randal Bollinger et al., 2003). This combination of polyreactivity and polyvalency provides natural antibodies with the capacity to bind and neutralize the multitude of different bacteria in the gut to help maintain the healthy relationship between commensal microbiota and host (Bunker et al., 2017). However, it also has potential significance for the B-lymphocyte response to stroke as at least one of the antigens that IgA natural antibodies are known to recognize are components of the cellular debris present within chronic stroke infarcts. This is because enzymatic and non-enymatic lipid peroxidation is common after brain injury, leading to the generation of oxidized phoshoplipids (Anthonymuthu et al., 2018). Additionally, we recently demonstrated that chronic stroke infarcts contain foamy macrophages which form in response to the excessive uptake of oxidized phospholipids on particles of low-density lipoprotein (Ahotupa, 2017; Chung et al., 2018), and which express oxidized phospholipids on their surface when they become apoptotic (Binder et al., 2005).

Therefore, the goal of this study was to learn more about the IgA response to stroke. To achieve this goal, we evaluated the kinetics of the appearance of B-lymphocytes, T-lymphocytes, and IgA + PCs within the infarct after stroke and determined if the IgA + PCs mature by a T-lymphocyte dependent or independent mechanism. We discovered that IgA + PCs, and the majority of the T- and B-lymphocytes that respond to stroke, do not appear within the infarct until several weeks after stroke. We discovered that the delayed infiltration of B-lymphocytes and IgA + PCs into the infarct following stroke can occur independently of CD4 T-lymphocyte help, and that IgA + PC maturation following stroke does not require MyD88 signaling. MyD88 signaling is a hallmark of a response to a thymus independent type 1 (TI-1) antigen (Boes, 2000; Hanihara-Tatsuzawa et al., 2014), which indicates that a population of IgA + PCs develop following stroke in response to a TI-2 antigen. Furthermore, sequencing the mRNA from single IgA + PCs present within the infarct revealed the presence of somatic hypermutation but not in the antigen-binding complementarity determining regions that typically indicate affinity maturation. These data provide additional evidence that a population of IgA + PCs develops in response to one or more TI-2 antigens (Adderson et al., 1998; Scheeren et al., 2008). These findings reveal that the production of IgA natural antibodies is part of the B-lymphocyte response to stroke.

## 2. Methods

### 2.1. Mice

Adult 3–4 month old male wildtype (WT) C57BL/6J, MHCII^−/−^ (B6.129S2-*H2dlAb1-Ea*/J Stock No: 003584), CD4^−/−^ (B6.129S2-*Cd4tm1Mak*/J Stock No: 002663), and MyD88^−/−^ (originally generated by Adachi et al. (Adachi et al., 1998)) mice were used for the study. Mice were purchased from Jackson Laboratory, with the exception of the MyD88^−/−^ mice which were bred at the University of Arizona and genotyped by tail biopsy. The lack of CD4 and MHCII expression in CD4^−/−^ and MHCII^−/−^ mice were confirmed through flow cytometric analysis. Mice were given food and water *ad libitum* while housed under a 12-hour light/dark schedule. All experiments were approved by the University of Arizona Institutional Animal Care and Use Committee and in accordance with the guidelines set by the National Institute of Health. Mice were euthanized at each time point through the use of isoflurane (JD Medical) anesthesia, exsanguination, and intracardial perfusion with 0.9% saline. Whole brains were then removed and placed in a 4% paraformaldehyde (PFA) solution for 24 h before being transferred into a 30% sucrose solution for immunostaining or were dissected immediately for flow cytometry analysis.

### 2.2. Stroke surgeries

Distal middle cerebral artery occlusion (DMCAO) plus hypoxia (DH stroke) was performed on all mice, as previously described (Doyle et al., 2012; Nguyen et al., 2016). Mice were anesthetized by isoflurane inhalation and an incision was made to expose the temporalis muscle. A pocket was created in the muscle to expose the skull and the right middle cerebral artery (MCA) was identified. A microdrill was then used to expose the underlying blood vessel. The meninges were cut and the vessel was cauterized using a small vessel cauterizer. The wound was then closed using surgical glue. Immediately following surgery, mice were placed in a hypoxia chamber containing 9% oxygen and 91% nitrogen for 45 min. Core body temperature was maintained at 37 °C throughout surgery, using a temperature controlled heating pad, and throughout hypoxia, using a heater within the chamber. Mice were given buprenorphine 0.1 mg/kg subcutaneously immediately following surgery and were given slow-release buprenorphine 1 mg/kg 24 h later. The animals also received cefazolin 25 mg/kg subcutaneously immediately following surgery. The mortality of the surgery was less than 5% and 0% mortality was observed up to 7 weeks following stroke. The DH stroke model creates a large infarct comprising approximately 25% of the ipsilateral hemisphere, has little variability, and has exceptional long-term survivability (Doyle et al., 2012). Hypoxia is necessary in this model because C57BL/6J mice that undergo DH stroke without hypoxia have significantly smaller infarcts (Doyle et al., 2012).

### 2.3. Immunostaining

A freezing sliding microtome (Microm HM 450, Thermo Fisher Scientific) was used to generate coronal sections (40 μm) spanning the infarct (Bregma 0.74 mm – 2.54 mm). Immunostaining was performed on free-floating brain sections using standard techniques (Doyle et al., 2015; Nguyen et al., 2016; Zbesko et al., 2018). The following primary antibodies were used: B220/CD45R (BD Biosciences, Cat. No. 553085, RRID: AB394615), CD3ε (BD Biosciences, Cat. No. 550277, RRID: AB393573), immunoglobulin A (IgA; BioLegend, Cat. No. 407004, RRID: AB315079), and CD138 (Syndecan-1;BioLegend, Cat. No. 142514, RRID: AB2562198). Sections were then labeled with the appropriate secondary antibody in conjunction with ABC Vector Elite and 3,3′-diaminobenzidine kits (Vector Laboratories) for visualization. For fluorescence imaging, sections were incubated in appropriate Alexa Fluor secondary antibodies (Thermo Fisher Scientific). Sections were imaged using a digital Keyence BZ-X700 light and fluorescent microscope or a Leica SP5-II laser scanning confocal microscope.

### 2.4. Image analysis

The number of cells and total percent area stained were analyzed using ImageJ analysis software (National Institutes of Health). For both methods, the area of the infarct was first measured by outlining the infarct for each section. For counting of total number of positive cells, the number of cells stained was divided by area of the infarct to obtain the final value. For total percent area stained, images were converted into 8-bit before utilizing the threshold function to obtain the final values. A minimum of three different sections were scored for each mouse and averaged.

### 2.5. Flow cytometry

Infarcts were visually identified and extracted from mice at 7 weeks post-stroke and processed to a single cell suspension by manual dissociation between the frosted ends of two microscope slides. Infarcts can be visually identified at 7 weeks due to a clear difference in color and visual texture compared to the rest of the brain as show in (Chung et al., 2018). Flow cytometry was performed as previously reported (Stokes et al., 2016). To minimize non-specific staining, cells were incubated with anti-mouse CD16/32 (BD Biosciences) for 15 min. Following this, cells were incubated with Live/Dead dye (Thermo Fisher Scientific, L-23105) and then antibodies against CD45 (BD Biosciences, Cat. No. 565478, RRID: AB2739257), MHCII (BioLegend, Cat. No.107611, RRID: AB313326), B220 (BioLegend, Cat. No. 103248, RRID: AB2650679), CD138 (BioLegend, Cat. No.142514, RRID: AB2562198), and CD3ε (BD Biosciences, Cat. No. 562286, RRID: AB11153307) for 30 min. Fluorescence data was collected using an LSRFortessa cell analyzer (BD Biosciences). Data were analyzed using FlowJo 2 (Tree Star) and all gating was based on fluorescence minus one (FMO) controls.

### 2.6. IgA mRNA sequencing

IgA + CD138 + PCs were single cell sorted from the infarct at 7 weeks post-stroke using the methodology described above with the addition of an antibody against IgA (Thermo Fisher Scientific, Cat. No. 11–4204-81, RRID: AB465220). Cells were isolated from WT mice using a FACSAria III (BD Biosciences) into a 96-well plate containing catch buffer and were then stored at −80 °C. Catch buffer contained 0.1 M Tris pH 8.0 and RNase inhibitor (Promega N2515). cDNA was then generated for each PC and the V(D)J regions were amplified by two rounds of nested PCR using primers and standard techniques (Table 1) (Ho et al., 2016; Tiller et al., 2009). The amplified regions were sequenced (Eton Biosciences). Sequences were visualized in SnapGene (GSL Biotech LLC) and compared to reference sequences for C57BL/6 mice using IgBLAST (NCBI). Specific V(D)J gene segments were analyzed for mutations if they had at least a 98% similarity with reference sequences. Mutations in the complementarity-determining regions (CDRs), as well as total mutations were calculated.

### 2.7. Statistical analysis

IHC and flow cytometric analysis were performed with blinding to experimental group and time point. Statistical analyses were performed using Prism 6.0 software (GraphPad) assuming all data was normally distributed. Data are expressed as mean with error bars indicating standard error of the mean (SEM). Each individual statistical test performed is indicated in the figure legend. A p-value of < 0.05 was considered significant. Sample size was determined using *a priori* power analysis using effect sizes based on previous experiments in the lab. Animals were excluded from analysis if there was no visible stroke infarct at the time of harvest, indicating a failed stroke surgery. Animals were randomized before surgery into set time points for harvest. Each independent experiment had its own WT control group.

## 3. Results

### 3.1. The appearance of B-lymphocytes, T-lymphocytes, and IgA + PCs within the infarct following stroke is delayed

**Fig. 1.**
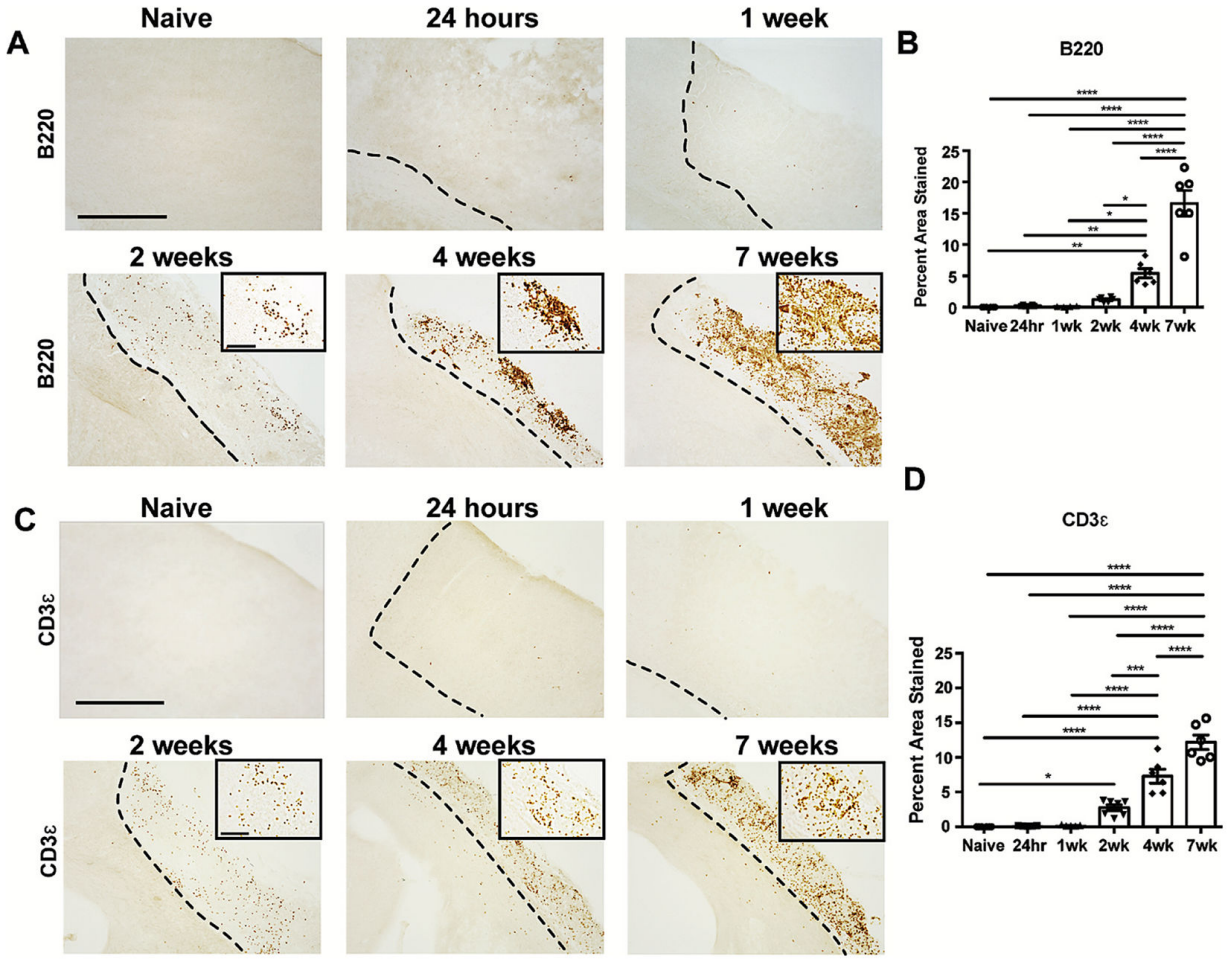
B- and T-Lymphocyte infiltration into the infarct following stroke is delayed. **A.)** Representative images of anti-B220 immunostaining of brains sections from naïve mice and mice 24 hours, 1 week, 2 weeks, 4 weeks, and 7 weeks following DH stroke. Images are taken at 10x and insets are 40x. Scale bar, 500 μm 10x and 150 μm 40x. **B.)** Percent area stained by B220 within the stroke infarct at 24 hours, 1 week, 2 weeks, 4 weeks, and 7 weeks following DH stroke. B-lymphocytes first appear within the infarct at 2 weeks post-stroke, and their numbers are significantly increased at 4 weeks and 7 weeks. **C.)** Representative images of anti-CD3ε immunostaining of brains sections from naïve mice and mice 24 hours, 1 week, 2 weeks, 4 weeks, and 7 weeks following DH stroke. Images are taken at 10x and insets are 40x. Scale bar, 500 μm 10x and 150 μm 40x. **D.)** Percent area stained by CD3ε within the stroke infarct at 24 hours, 1 week, 2 weeks, 4 weeks, and 7 weeks following DH stroke. T-lymphocytes are significantly increased at 2 weeks, 4 weeks, and 7 weeks following stroke. n = 5–7 per group. Data represent mean ± SEM. *p < 0.05, **p < 0.01, ***p < 0.001, ****p < 0.0001 by one-way ANOVA with comparisons against all other groups, with post-hoc Tukey’s multiple comparisons test.

We previously demonstrated that there is a B-lymphocyte response within the infarct at 7 weeks following stroke that contributes to delayed cognitive impairment (Chung et al., 2018; Doyle et al., 2015; Zbesko et al., 2018). However, the temporal kinetics of B-lymphocyte infiltration into the brain were still unknown. Therefore, to map the arrival and accumulation of B-lymphocytes in the brain following stroke, we evaluated B220 immunoreactivity in the brains of naïve mice and mice sacrificed at 24 hours, 1 week, 2 weeks, 4 weeks, and 7 weeks following stroke (Fig. 1A). B-lymphocytes first appeared within the infarct at 2 weeks following stroke. Their numbers were significantly increased at 4 weeks and continued to climb between 4 weeks and 7 weeks following stroke (Fig. 1B). To determine if T-lymphocytes follow a similar infiltration pattern, we also stained the naïve mice, as well as the mice sacrificed at 24 hours, 1 week, 2 weeks, 4 weeks, and 7 weeks following stroke for CD3ε (Fig. 1C). T-lymphocytes were significantly increased in the infarct at 2 weeks following stroke, and their infiltration was also progressively increased at 4 weeks and 7 weeks following stroke (Fig. 1D).

**Fig. 2.**
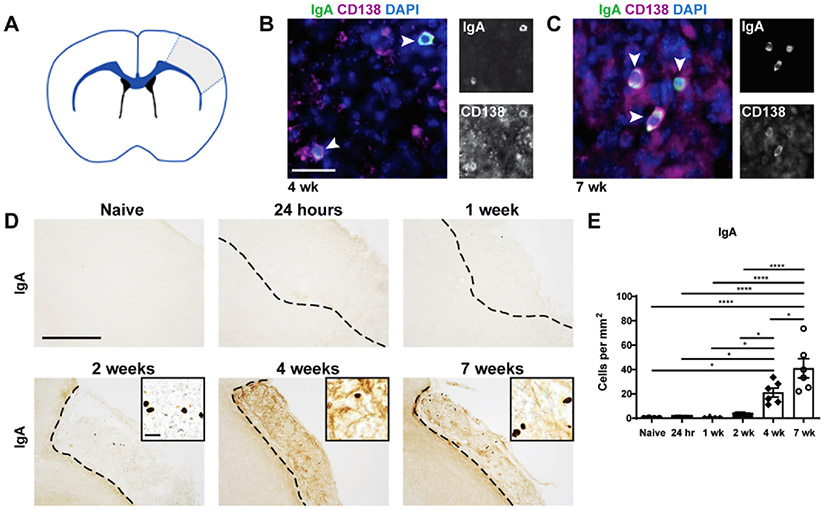
PCs take multiple weeks to appear within the stroke infarct. **A.)** A schematic representation of the infarcted brain, with gray indicating the area of damage and, thus, the area of quantification **B&C.)** Representative images of immunofluorescent staining with anti-IgA (green), anti-CD138 (purple), and DAPI (blue) showing colocalization of CD138, a plasma cell marker, with IgA at 4 (**B**) and 7 weeks post-stroke (**C**). Scale bar, 25 μm. n = 3. **D.)** Representative images of anti-IgA immunostaining of brains sections from naïve mice and mice 24 hours/, 1 week, 2 weeks, 4 weeks, and 7 weeks following DH stroke. Images are taken at 10x and insets are 40x. Scale bar, 500 μm 10x and 25 μm 40x. **E.)** Quantification of the number of IgA + cells per mm^2^ of stroke infarct at each time point post-stroke. IgA + plasma cells enter the stroke infarct between 2 and 4 weeks post-stroke and persist for at least 7 weeks post-stroke. Data represent mean ± S.E.M. *p < 0.05, ****p < 0.0001 by one-way ANOVA with comparisons against all other groups, with post-hoc Tukey’s multiple comparisons test. n = 5–7.

We also previously demonstrated that IgA + cells are present within the infarct at 7 weeks following stroke (Doyle et al., 2015). To confirm that these IgA + cells are antibody-producing PCs, we performed immunofluorescent (IF) staining for IgA and CD138, a marker of immunoglobulin-synthesizing PCs (Fig. 2). The IgA + cells within the infarct at 4 and 7 weeks following stroke co-localized with CD138. This confirms that the IgA + cells are antibody-producing PCs (Fig. 2B&C). To determine the temporal kinetics of IgA + PC appearance within the infarct, immunohistochemistry (IHC) was performed on brain tissue from naïve mice and mice sacrificed at 24 hours, 1 week, 2 weeks, 4 weeks, and 7 weeks post-stroke (Fig. 2D). IgA + PCs were significantly increased in number at 4 weeks and 7 weeks post-stroke compared to naïve controls (Fig. 2E).

Together, these data demonstrate that the appearance of B-lymphocytes and IgA + PCs in the infarct following stroke is delayed, with B-lymphocyte and IgA + PC numbers not peaking until several weeks after stroke.

### 3.2. A population of B-lymphocytes mature into IgA + PCs through a T-lymphocyte independent mechanism

**Fig. 3.**
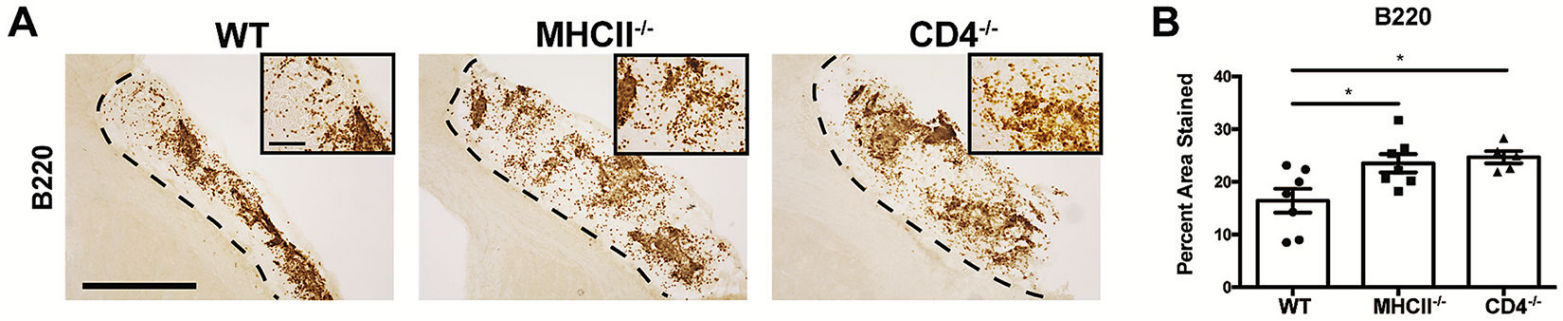
A B-lymphocyte response to stroke still occurs in CD4^−/−^ and MHCII^−/−^ mice. **A.)** Representative images of anti-B220 immunostaining within the infarct of brain sections from WT, MHCII^−/−^, and CD4^−/−^ mice at 7 weeks post-DH stroke. Images are taken at 10x and insets are 40x. Scale bar, 500 μm 10x and 100 μm 40x. **B.)** Quantification of the percent area stained reveals there are significantly more B-lymphocytes within the infarct of MHCII^−/−^ and CD4^−/−^ mice compared to WT mice at 7 weeks post-DH stroke. Data represent mean ± SEM. *p < 0.05 by one-way ANOVA. n = 5–7 mice per group.

Isotype switching is a critical step in the maturation of B-lymphocytes into IgA + PCs, and the ability to isotype switch into IgA can occur through either a CD4 T-lymphocyte dependent or a CD4 T-lymphocyte independent mechanism (Fagarasan et al., 2010). To determine which of these mechanisms results in the maturation of B-lymphocytes into IgA + PCs following stroke, we performed stroke surgery on WT, CD4^−/−^, and MHCII^−/−^ mice. IHC at 7 weeks following stroke revealed no decrease in the number of B220 + B-lymphocytes within the infarct of the CD4^−/−^ and MHCII^−/−^ mice (Fig. 3A). Rather, there was an increase in the number of B220 + B-lymphocytes in both strains of transgenic mice compared to WT controls (Fig. 3B). This demonstrates that a B-lymphocyte response to stroke still occurs in CD4^−/−^ and MHCII^−/−^ mice.

**Fig. 4.**
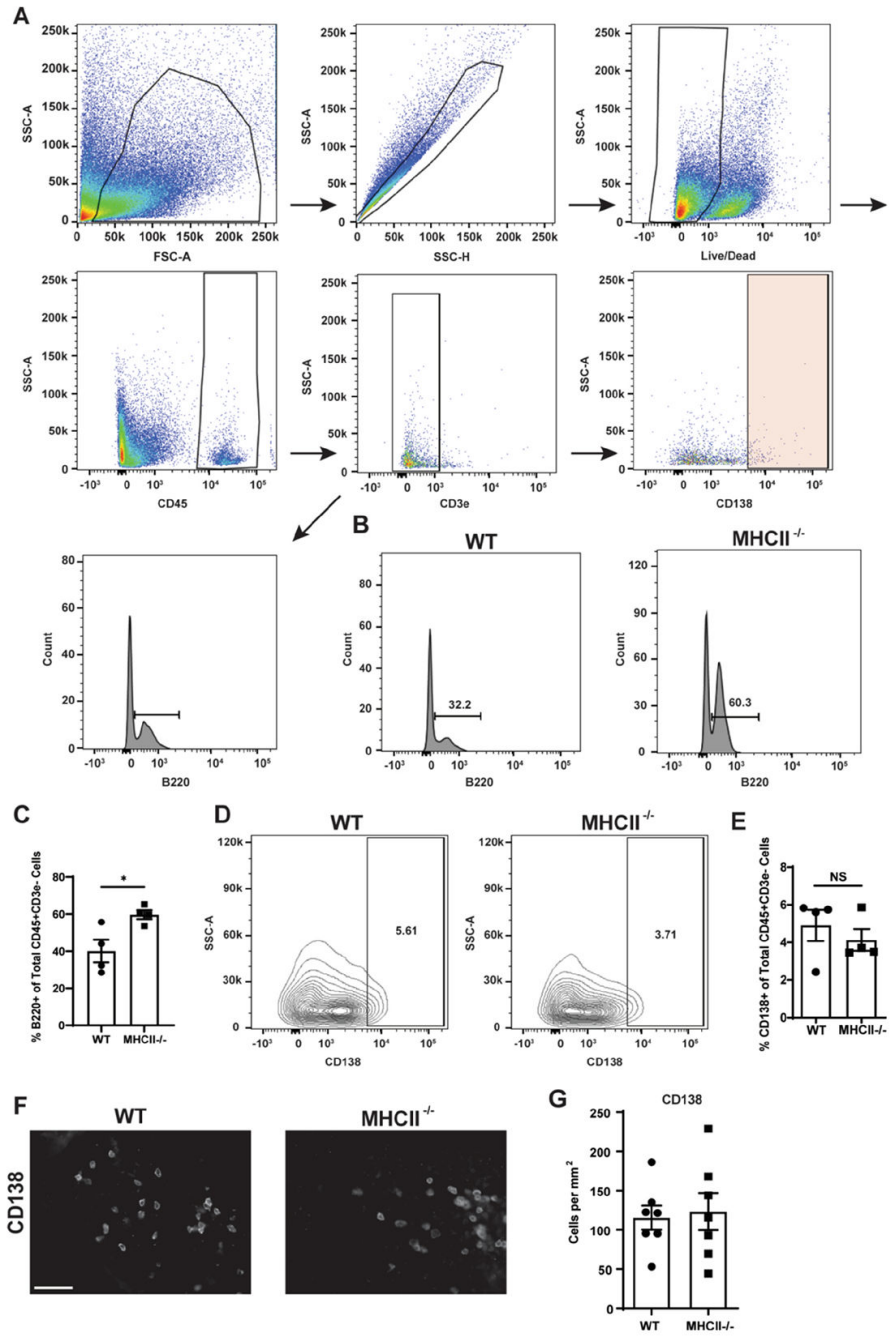
PCs are present within the infarcts of MHCII^−/−^ mice. **A.)** Flow cytometry was performed on the infarcts of mice 7 weeks post-stroke. PCs were gated first by forward (FSC) and side (SSC) scatter to eliminate debris and then SSC area and SSC height to exclude doublets. Live/dead staining was then used to exclude dead cells. CD45 + staining was used to identify lymphocytes. CD3ε + cells (T-lymphocytes) were then excluded. B220 + or CD138 + was then used to determine the percentage of B-lymphocytes or PCs, respectively, in the remaining population. **B.)** Representative flow cytometry plots from the two mouse strains reveal an increased percentage of B-lymphocytes present within the CD45 + CD3ε-population in the infarct at 7 weeks post-DH stroke in MHCII^−/−^ mice. **C.)** Quantification of the flow cytometry analysis of the percent B220 + cells out of the total CD45 + CD3ε- population. **D.)** Representative flow cytometry plots from the two mouse strains reveal a similar percentage of PCs present within the CD45 + CD3ε- population in the infarct of both WT and MHCII^−/−^ mice at 7 weeks post-DH stroke. **E.)** Quantification of the flow cytometry analysis of the percent CD138 + cells out of the total CD45 + CD3ε- population. **F.)** Representative images of anti-CD138 immunostaining of brains sections from WT and MHCII^−/−^ mice at 7 weeks post-DH stroke. Images are taken at 40x. Scale bar 100 μm. **G.)** Quantification of the number of CD138 + cells per mm^2^ in each of the strains revealed no difference between them in the number of PCs present within the infarct. n = 4 mice per group (flow cytometry), n = 7 mice per group (immunofluorescence). Data represent mean ± SEM. *p < 0.05 by Student’s *t* test.

To further investigate the contribution of CD4 T-lymphocytes to the B-lymphocyte response to stroke, WT and MHCII^−/−^ mice underwent experimental stroke and, at 7 weeks following stroke, the abundance of CD138 + PCs in the infarct was quantified by flow cytometry and immunofluorescent staining. Following the flow cytometry gating scheme shown in Fig. 4A, we observed an increased percentage of B220 + cells in the infarcts of the MHCII^−/−^ mice (Fig. 4B&C), consistent with our finding in Fig. 3B. We observed no difference in the percentage of PCs in the infarcts from the WT and MHCII^−/−^ mice at 7 weeks following stroke (Fig. 4D&E). Additionally, we observed no difference in the number of CD138 + PCs in the infarct at 7 weeks following stroke by immunofluorescence (Fig. 4F&G). These data demonstrate that following stroke, there is a population of PCs within the infarct that develops through a CD4 T-lymphocyte independent mechanism.

**Fig. 5.**
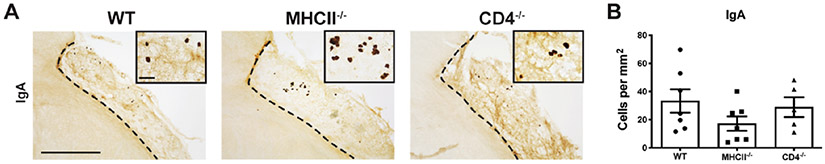
IgA + PCs appear in the infarct independently of T-lymphocytes. **A.)** Representative images of anti-IgA immunostaining of brains sections from WT, MHCII^−/−^, and CD4^−/−^ mice at 7 weeks post-DH stroke. Images are taken at 10x and insets are 40x. Scale bar, 500 μm 10x and 150 μm 40x. **B.)** Quantification of the number of IgA + cells per mm^2^ in each of the three strains of mice indicates that the number of PCs present within the infarct of each strain is similar. Data represent mean ± SEM. Not significant by one-way ANOVA. n = 5–7 mice per group.

We next sought to determine if the population of PCs that develops through a CD4 T-lymphocyte independent mechanism includes IgA + PCs. WT, CD4^−/−^, and MHCII^−/−^ mice underwent experimental stroke and the number of IgA + PCs present in the infarct at 7 weeks following stroke was quantified by IHC (Fig. 5A). There was no significant difference in the number of IgA + cells within the infarcts of the CD4^−/−^ and MHCII^−/−^ mice compared to WT mice (Fig. 5B). These data indicate that the IgA + PC response to stroke is CD4 T-lymphocyte independent.

### 3.3. A population of B-lymphocytes mature into IgA + PCs following an interaction with a TI-2 antigen

The activation of B-lymphocytes in the absence of help from CD4 + T-lymphocytes can only occur in response to a thymus-independent type 1 (TI-1) antigen or a thymus-independent type 2 (TI-2) antigen (Parker, 1993). A TI-1 antigen is able to directly induce the proliferation and differentiation of B-lymphocytes through high levels of myeloid differentiation primary response 88 (MyD88) signaling, normally through the toll like receptors (TLRs) expressed by the B-lymphocyte, thus not requiring the B-cell receptor (BCR) on the B-lymphocyte to have specificity to the antigen (Boes, 2000; Hanihara-Tatsuzawa et al., 2014). TI-2 antigens, on the other hand, require specificity towards the BCR of the B-lymphocyte, as they are molecules containing long, repeating epitopes that crosslink multiple BCRs on the surface of the cell (Allman et al., 2019; Haas and Estes, 2000). To determine if the IgA + PC response to stroke is in response to a TI-1 or a TI-2 antigen, we performed stroke surgery on WT and MyD88^−/−^ mice.

**Fig. 6.**
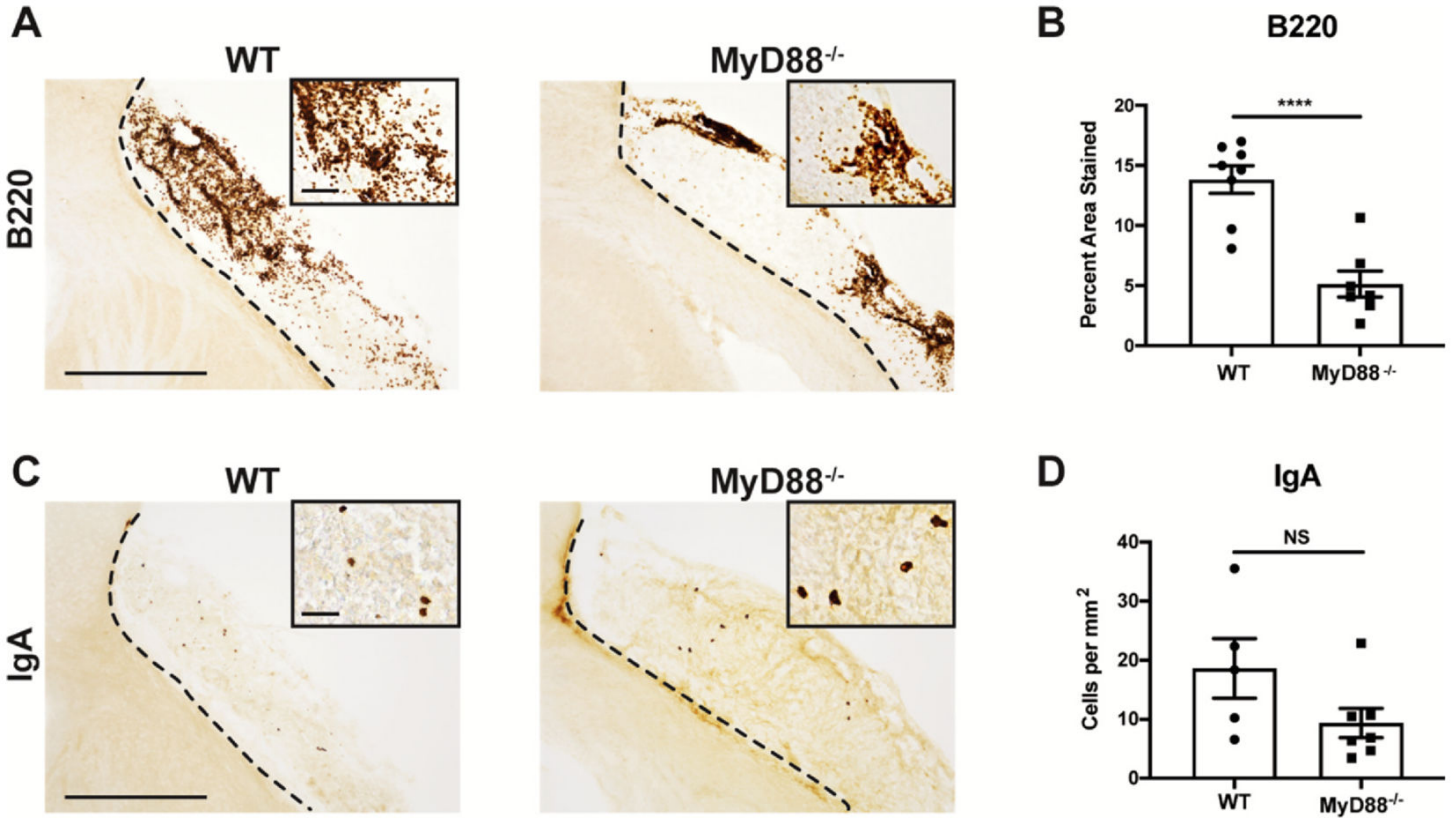
IgA + PCs can develop post-stroke following activation by a TI-2 antigen. **A.)** Representative images of anti-B220 immunostaining of brains sections from WT and MyD88^−/−^ mice at 7 weeks post-DH stroke. Images are taken at 10x and insets are 40x. Scale bar, 500 μm 10x and 150 μm 40x. **B.)** B220 percent area stained indicates that there are more B-lymphocytes in WT mice than MyD88^−/−^ mice. Data represent mean ± SEM. ****p < 0.0001 by Student’s T-test. n = 5–7 mice per group. **C.)** Representative images of anti-IgA immunostaining of brains sections from WT and MyD88^−/−^ mice at 7 weeks post-DH stroke. Images are taken at 10x and insets are 40x. Scale bar, 500 μm 10x and 100 μm 40x. **D.)** Quantification of the number of IgA + cells per mm^2^ shows there is no difference in the number of IgA + plasma cells between WT and MyD88^−/−^ mice. Data represent mean ± SEM. NS by Student’s T-test. n = 5–7 mice per group.

B220 immunoreactivity was measured in the infarct at 7 weeks following stroke in the two strains of mice (Fig. 6A). There was a significant decrease in the total percent area stained in the MyD88^−/−^ mice, but B-lymphocytes were still present (Fig. 6B). To investigate which type of antigen is responsible for the maturation of B-lymphocytes into IgA + PCs after stroke, we then performed IHC using an anti-IgA antibody (Fig. 6C). This revealed no significant difference in the number of IgA + PCs within the infarcts of the WT and MyD88^−/−^ mice, indicating that following stroke, a population of IgA + PCs develops following activation by a TI-2 antigen (Fig. 6D).

To investigate B-lymphocyte maturation into IgA + PCs following stroke in response to a TI-2 antigen further, we sequenced the antibody variable region from IgA + PCs isolated from the infarct at 7 weeks post-stroke (Table 2). We identified somatic hypermutation, indicated by the total number of mutations, but minimal affinity maturation, indicated by the low number of mutations in the CDRs, in 9 out of the 10 PCs sequenced. The presence of somatic hypermutation and lack of affinity maturation are characteristics of T-lymphocyte independent activation in response to a TI-2 antigen (Adderson et al., 1998; Scheeren et al., 2008).

## 4. Discussion

IgA + PCs are typically located within the gut of animals, where they help maintain the delicate balance between the host and its microbiome by producing either antigen-specific or natural polyreactive antibodies (Benckert et al., 2011; Bunker et al., 2017; Hoces et al., 2020). However, we recently published that IgA + cells are present within stroke infarcts at 7 weeks following stroke (Doyle et al., 2015). As these cells are typically not found in the brain, we focused our study on IgA + PCs to determine how these PCs develop following stroke and to learn more about their role in stroke recovery.

Our first objective was to determine when B-lymphocytes appear within the stroke infarct. In other models of stroke, B-lymphocytes have been observed to play a beneficial role as early as 24 hours post-stroke, although other studies report that B-lymphocytes do not influence stroke outcome in the acute phase (Ren et al., 2011; Schuhmann et al., 2017). Our study indicates that a more extensive B-lymphocyte response to stroke occurs within the infarct at a much later time point, beginning 2–4 weeks after stroke and peaking 7 weeks after stroke. This is in agreement with data from Vindegaard et al. in which they showed peak lymphocyte infiltration occurring 4 weeks after stroke, which was the final time point they investigated (Vindegaard et al., 2017). Our T-lymphocyte time course demonstrates that peak T-lymphocyte infiltration into the brain following stroke is also delayed, with numbers only significantly above naïve mice in our study at 2 weeks post-stroke, and progressively increasing until at least 7 weeks post-stroke.

The finding that adaptive immune cell infiltration does not peak until at least 4 weeks after stroke is further supported by the delayed appearance of IgA + PCs in the infarct. IgA + PCs begin to populate the infarct at 2 weeks following stroke, and their numbers are even higher at 4 weeks and 7 weeks after stroke. These data suggest that in addition to the early anti-inflammatory role that B-lymphocytes play in the acute phase of stroke, there is a second B-lymphocyte response to unidentified antigens during the chronic phase that features the maturation of activated B-lymphocytes into IgA + PCs.

Our data does not indicate whether the IgA + PCs present in the infarct at 4- and 7-weeks following stroke are developing in the infarct or are migrating from the periphery. However, Rojas et al have shown that in EAE, IgA + PCs translocate from the gut into the brain. These cells produce IL-10, which limits neuroinflammation and mitigates disease severity (Rojas et al., 2019). Thus, the IgA + PCs we observe may be migrating from the gut to perform an IL-10 dependent anti-inflammatory function, which would be in accordance with the findings of Rojas et al (Rojas et al., 2019). However, we have previously shown no difference in levels of IL-10 within the infarct at 7 weeks following stroke between WT and MuMT mice (Doyle et al., 2015). This makes it unlikely that IgA + PCs are contributing significantly to the pool of IL-10 in the stroke infarct at this chronic time point.

To increase our understanding of the IgA + PC response to stroke, we sought to determine the extent to which it is CD4 T-lymphocyte dependent and discovered that IgA + PCs appear in the infarct independently of CD4 T-lymphocyte help. Surprisingly, this experiment also revealed that the infiltration of B-lymphocytes within the infarct at 7 weeks post-stroke is significantly increased in MHCII^−/−^ and CD4^−/−^ mice compared to WT mice. This demonstrates that the delayed infiltration of B-lymphocytes into the infarct following stroke can also occur independently of CD4 T-lymphocyte help. These data indicate that a sizable fraction of the B-lymphocyte response to stroke is T-lymphocyte independent. However, they do not exclude that a T-lymphocyte dependent B-lymphocyte response occurs simultaneously in wildtype mice, which is in fact supported by Weitbrecht et al’s finding in this special issue that CD4 T cells also promote B cell responses after stroke (Weitbrecht et al., 2020). Notably, although we see an increase in B-lymphocytes in MHCII^−/−^ and CD4^−/−^ mice compared to WT mice, we do not see a corresponding increase in PCs in these mice, indicating that a smaller percentage of the B-lymphocytes present are maturing into PCs. This could be attributed to the altered cytokine milieu due to the lack of activated T-lymphocytes or the specificity of the additional B-lymphocytes to the activating antigen(s). While this is an area that needs more investigation, it does not detract from our finding that a population of IgA + PCs develops independently of T-lymphocytes following stroke.

Having determined that IgA + PCs mature through a T-lymphocyte independent mechanism following stroke, we next sought to determine whether the activating antigen was a TI-1 or TI-2 antigen. Using MyD88^−/−^ mice, which lack the ability to undergo TI-1-mediated T-lymphocyte independent B-lymphocyte activation, we observed that there was still a population of IgA + PCs within the infarct of the MyD88^−/−^ mice at 7 weeks following stroke. This demonstrates that a population of IgA + PCs develops following stroke in response to one or more TI-2 antigens. As a caveat, there was a downward trend in the number of IgA + PCs present in the infarcts of the MyD88^−/−^ mice. However, this may be due to the fact that the lack of MyD88 signaling also resulted in substantially fewer B-lymphocytes being present in the infarcts of the MyD88^−/−^ mice.

In further support of our finding that the maturation of a population of IgA + PCs following stroke is in response to, at least in part, one or more TI-2 antigens, we observed the presence of somatic hypermutation but a lack of affinity maturation within the mRNA sequenced from IgA + PCs directly isolated from the infarct. These characteristics are hallmarks of B-cell activation following stimulation with a TI-2 antigen (Adderson et al., 1998; Scheeren et al., 2008).

The role of IgA natural antibodies in stroke recovery is unknown. Natural antibodies have broad reactivity against self-antigens, many of which display molecular mimicry with foreign antigens. The most well-characterized self-antigen epitopes include oxidized phospholipids, oxidized low-density lipoprotein particles, glycolipids, and glycoproteins (Holodick et al., 2017), and the most common foreign epitopes include bacterial phosphorylcholine, LPS, and flagellin (Bunker et al., 2017; Holodick et al., 2017). Significantly, oxidized low-density lipoprotein mediates the transformation of macrophages to cholesterol-rich foam cells and we have previously demonstrated that chronic stroke infarcts contain foamy macrophages (Chung et al., 2018). A number of *in vitro* studies have provided evidence that natural antibodies block the uptake of oxidized low-density lipoprotein by macrophages and thus prevent foam cell formation (Binder et al., 2005, 2003; Hörkkö et al., 1999). Therefore, the production of natural antibodies following stroke may be a mechanism for counteracting the formation of foam cells within chronic stroke infarcts.

Additionally, apoptotic cell membranes contain oxidized phospholipids due to enhanced oxidative stress as a result of the loss of mitochondrial membrane integrity and the release of redox-active cytochrome c. These oxidation-specific epitopes are immunodominant epitopes on apoptotic cells and the binding of natural antibodies to oxidized phospholipids neutralizes their pro-inflammatory properties (Binder et al., 2005). Chronic stroke infarcts almost certainly contain foamy macrophages undergoing apoptosis, because at 4 weeks and 8 weeks following stroke these cells are laden with cholesterol crystals, which are potent inducers of programmed cell death via inflammasome signaling (Chung et al., 2018). Therefore, the production of natural antibodies following stroke may also be a mechanism for counteracting the pro-inflammatory properties of apoptotic cells within chronic stroke infarcts.

In summary, the data presented here demonstrate that IgA + PCs first begin to appear within stroke infarcts between 2- and 4-weeks following stroke and continue to accumulate for at least 3 more weeks. A population of these cells develops through a T-lymphocyte independent mechanism, likely through an interaction with one or more TI-2 antigens, and they secrete natural antibodies. However, future studies are required to determine if one of the functions of these natural antibodies is to assist in the clearance of myelin debris and neutralization of apoptotic foamy macrophages.

## Figures and Tables

**Table 1:** Primers used to amplify VD(J) regions of PCs. Two rounds of nested PCR were performed on cDNA generated from each PC.

1st Round Primers		2nd Round Primers	
HC PCR		HC PCR	
Primer Name	Primer sequence	Primer Name	Primer sequence
5′ MsVHE	GGGATTCGAGGTGCAGCTGCAGGAGTCTGG	5′ MsVHE	GGGATTCGAGGTGCAGCTGCAGGAGTCTGG
3′ Cα outer(IgA)	GGAAGTTTACGGTGGTTATATCC	3′ Cα inner(IgA)	TGCCGGAAGGGAAGTAATCGTGAAT
Kappa PCR		Kappa PCR	
Primer Name	Primer Sequence	Primer Name	Primer Sequence
5′ VK3	TGCTGCTGCTCTGGGTTCCAG	5′ mVkappa	GAYATTGTGMTSACMCARWCTMCA
5′ VK4	ATTWTCAGCTTCCTGCTAATC	3′ BsiWI P-mJK01	GCCACCGTACGTTTGATTTCCAGCTTGGTG
5′ VK5	TTTTGCTTTTCTGGATTYCAG	3′ BsiWI P-mJK02	GCCACCGTACGTTTTATTTCCAGCTTGGTC
5′ VK6	TCGTGTTKCTSTGGTTGTCTG	3′ BsiWI P-mJK03	GCCACCGTACGTTTTATTTCCAACTTTGTC
5′ VK689	ATGGAATCACAGRCYCWGGT	3′ BsiWI P-mJK04	GCCACCGTACGTTTCAGCTCCAGCTTGGTC
5′ VK14	TCTTGTTGCTCTGGTTYCCAG		
5′ VK19	CAGTTCCTGGGGCTCTTGTTGTTC		
5′ VK20	CTCACTAGCTCTTCTCCTC		
3′MCK	GATGGTGGGAAGATGGATACAGTT		

**Table 2:** The IgA sequences from IgA + PCs isolated from the infarct 7 weeks post-DH stroke. IgA + PCs were single cell sorted by gating of live CD45 + cells and then sorting CD138 + IgA + cells. Shown are 10 sequences of the mRNA encoding IgA from isolated PCs. The V, D, and J genes are shown for both the light and heavy chain. The number of mutations within the complementarity-determining regions (CDRs) and total number of mutations are also shown. n = 4 mice from which 10 IgA + PCs were sequenced.

Clone	Chain	V	D	J	CDR mutations	Total Mutations
B1	Light	IGKV14-111*01		IGKJ5*01	4	8
B1	Heavy	IGHV1-9*01	IGHD2-3*01	IGHJ4*01	2	25
B2	Light	IGKV3-5*01		IGKJ1*01	8	30
B2	Heavy	IGHV1S52*01	IGHD4-1*02	IGHJ4*01	6	26
B3	Light	IGKV4-59*01		IGKJ1*01	0	5
B3	Heavy	IGHV1-54*01	IGHD2-5*01	IGHJ2*01	3	10
B4	Light	IGKV17-127*01		IGKJ2*01	2	4
B4	Heavy	IGHV14-3*01	IGHD1-3*01	IGHJ1*01	2	6
B5	Light	IGKV8-19*01		IGKJ4*01	1	2
B5	Heavy	IGHV1-54*01	IGHD2-5*01	IGHJ2*01	9	29
B10	Light	IGKV3-4*01		IGKJ4*01	0	1
B10	Heavy	IGHV1-20*01	IGHD1-1*01	IGHJ1*03	2	12
B11	Light	IGKV3-5*01		IGKJ1*01	1	6
B11	Heavy	IGHV1-53*01	IGHD4-1*01	IGHJ3*01	0	3
C4	Light	IGKV19-93*01		IGKJ5*01	0	3
C4	Heavy	IGHV1-63*01	IGHD3-2*01	IGHJ2*01	1	14
C6	Light	IGKV3-4*01		IGKJ1*01	0	4
C6	Heavy	IGHV1S74*01	IGHD1-1*01	IGHJ3*01	1	5
D3	Light	IGKV3-4*01		IGKJ1*01	0	14
D3	Heavy	IGHV1-58*01	IGHD6-1*01	IGHJ3*01	0	5

## References

[R1] Adachi O, Kawai T, Takeda K, Matsumoto M, Tsutsui H, Sakagami M, Nakanishi K, Akira S, 1998. Targeted disruption of the MyD88 gene results in loss of IL-1- and IL- 18-mediated function. Immunity. 10.1016/S1074-7613(00)80596-8. 9697844

[R2] Adderson EE, Shackelford PG, Carroll WL, 1998. Somatic hypermutation in T-independent and T-dependent immune responses to Haemophilus influenzae type B polysaccharide. Clin. Immunol. Immunopathol 10.1006/clin.1998.4603. 9837694

[R3] Ahotupa M, 2017. Oxidized lipoprotein lipids and atherosclerosis. Free Radic. Res 10.1080/10715762.2017.1319944. 28412863

[R4] Allman D, Wilmore JR, Gaudette BT, 2019. The continuing story of T-cell independent antibodies. Rev Immunol. 10.1111/imr.12754. PMC6653682 30874357

[R5] Anthonymuthu TS, Kenny EM, Lamade AM, Kagan VE, Bayır H, 2018. Oxidized phospholipid signaling in traumatic brain injury. Biol. Med Free Radic 10.1016/j.freeradbiomed.2018.06.031. PMC6098726 29964171

[R6] Benckert J, Schmolka N, Kreschel C, Zoller MJ, Sturm A, Wiedenmann B, Wardemann H, 2011. The majority of intestinal IgA + and IgG + plasmablasts in the human gut are antigen-specific. J. Clin. Invest 10.1172/JCI44447. PMC3083800 21490392

[R7] Binder CJ, Hörkkö S, Dewan A, Chang MK, Kieu EP, Goodyear CS, Shaw PX, Palinski W, Witztum JL, Silverman GJ, 2003. Pneumococcal vaccination decreases atherosclerotic lesion formation: Molecular mimicry between Streptococcus pneumoniae and oxidized LDL. Med Nat. 10.1038/nm876. 12740573

[R8] Binder CJ, Shaw PX, Chang MK, Boullier A, Hartvigsen K, Hörkkö S, Miller YI, Woelkers DA, Corr M, Witztum JL, 2005. The role of natural antibodies in atherogenesis. J. Lipid Res 10.1194/jlr.R500005-JLR200. 15897601

[R9] Boes M, 2000. Role of natural and immune IgM antibodies in immune responses. Immunol Mol. 10.1016/S0161-5890(01)00025-6. 11451419

[R10] Bunker JJ, Erickson SA, Flynn TM, Henry C, Koval JC, Meisel M, Jabri B, Antonopoulos DA, Wilson PC, Bendelac A, 2017. Natural polyreactive IgA antibodies coat the intestinal microbiota. Science (80-.) 10.1126/science.aan6619. PMC5790183 28971969

[R11] Chung AG, Frye JB, Zbesko JC, Constantopoulos E, Hayes M, Figueroa AG, Becktel DA, Antony Day W, Konhilas JP, McKay BS, Nguyen TVV, Doyle KP, 2018. Liquefaction of the brain following stroke shares a similar molecular and morphological profile with atherosclerosis and mediates secondary neurodegeneration in an osteopontin-dependent mechanism. eNeuro. 10.1523/ENEURO.0076-18.2018. PMC6223114 30417081

[R12] Donaldson GP, Ladinsky MS, Yu KB, Sanders JG, Yoo BB, Chou WC, Conner ME, Earl AM, Knight R, Bjorkman PJ, Mazmanian SK, 2018. Gut microbiota utilize immunoglobulin a for mucosal colonization. Science 80-, ). 10.1126/science.aaq0926. PMC5973787 29724905

[R13] Doyle KP, Fathali N, Siddiqui MR, Buckwalter MS, 2012. Distal hypoxic stroke: A new mouse model of stroke with high throughput, low variability and a quantifiable functional deficit. J. Neurosci. Methods 10.1016/j.jneumeth.2012.03.003. PMC3348433 22465679

[R14] Doyle KP, Quach LN, Solé M, Axtell RC, Nguyen TVV, Soler-Llavina GJ, Jurado S, Han J, Steinman L, Longo FM, Schneider JA, Malenka RC, Buckwalter MS, 2015. B-lymphocyte-mediated delayed cognitive impairment following stroke. J. Neurosci 10.1523/JNEUROSCI.4098-14.2015. PMC4315838 25653369

[R15] Fagarasan S, Kawamoto S, Kanagawa O, Suzuki K, 2010. Adaptive Immune Regulation in the Gut: T Cell-Dependent and T Cell-Independent IgA Synthesis. Rev. Immunol Annu 10.1146/annurev-immunol-030409-101314. 20192805

[R16] Haas KM, Estes DM, 2000. Activation of bovine B cells via surface immunoglobuIin M cross-linking or CD40 ligation results in different B-cell phenotypes. Immunology. 10.1046/j.1365-2567.2000.00962.x. PMC2327142 10692047

[R17] Hanihara-Tatsuzawa F, Miura H, Kobayashi S, Isagawa T, Okuma A, Manabe I, MaruYama T, 2014. Control of Toll-like receptor-mediated T cell-independent type 1 antibody responses by the inducible nuclear protein IκB-ζ. J. Biol. Chem 10.1074/jbc.M114.553230. PMC4223300 25124037

[R18] Ho IY, Bunker JJ, Erickson SA, Neu KE, Huang M, Cortese M, Pulendran B, Wilson PC, 2016. Refined protocol for generating monoclonal antibodies from single human and murine B cells. J. Immunol. Methods 10.1016/j.jim.2016.09.001. PMC5322767 27600311

[R19] Hoces D, Arnoldini M, Diard M, Loverdo C, Slack E, 2020. Growing, evolving and sticking in a flowing environment: understanding IgA interactions with bacteria in the gut. Immunology. 10.1111/imm.13156. PMC6904610 31777063

[R20] Holodick NE, Rodríguez-Zhurbenko N, Hernández AM, 2017. Defining natural antibodies. Immunol Front. 10.3389/fimmu.2017.00872. PMC5526850 28798747

[R21] Hörkkö S, Bird DA, Miller E, Itabe H, Leitinger N, Subbanagounder G, Berliner JA, Friedman P, Dennis EA, Curtiss LK, Palinski W, Witztum JL, 1999. Monoclonal autoantibodies specific for oxidized phospholipids or oxidized phospholipid-protein adducts inhibit macrophage uptake of oxidized low-density lipoproteins. J. Clin. Invest 10.1172/JCI4533. PMC407862 9884341

[R22] Mishima Y, Oka A, Liu B, Herzog JW, Eun CS, Fan TJ, Bulik-Sullivan E, Carroll IM, Hansen JJ, Chen L, Wilson JE, Fisher NC, Ting JPY, Nochi T, Wahl A, Victor Garcia J, Karp CL, Balfour Sartor R, 2019. Microbiota maintain colonic homeostasis by activating TLR2/MyD88/PI3K signaling in IL-10-producing regulatory B cells. J. Clin. Invest 10.1172/JCI93820. PMC6715367 31211700

[R23] Nguyen TVV, Frye JB, Zbesko JC, Stepanovic K, Hayes M, Urzua A, Serrano G, Beach TG, Doyle KP, 2016. Multiplex immunoassay characterization and species comparison of inflammation in acute and non-acute ischemic infarcts in human and mouse brain tissue. Acta Neuropathol. Commun 4, 100. 10.1186/s40478-016-0371-y. 27600707 PMC5011964

[R24] Ortega SB, Noorbhai I, Poinsatte K, Kong X, Anderson A, Monson NL, Stowe AM, 2015. Stroke induces a rapid adaptive autoimmune response to novel neuronal antigens. Discov Med 19, 381–392. 26105701 PMC4692161

[R25] Palma J, Tokarz-Deptuła B, Deptuła J, Deptuła W, 2018. Natural antibodies – Facts known and unknown. Eur. J. Immunol Cent 10.5114/ceji.2018.81354. PMC6384419 30799995

[R26] Parker DC, 1993. T Cell-Dependent B Cell Activation. Rev. Immunol Annu 10.1146/annurev.iy.11.040193.001555. 8476565

[R27] Prencipe M, Ferretti C, Casini AR, Santini M, Giubilei F, Culasso F, 1997. Stroke, disability, and dementia: Results of a population survey. Stroke. 10.1161/01.STR.28.3.531. 9056607

[R28] Quan CP, Berneman A, Pires R, Avrameas S, Bouvet JP, 1997. Natural polyreactive secretory immunoglobulin A autoantibodies as a possible barrier to infection in humans. Immun Infect. 10.1128/iai.65.10.3997-4004.1997. PMC175574 9316998

[R29] Randal Bollinger R, Everett ML, Palestrant D, Love SD, Lin SS, Parker W, 2003. Human secretory immunoglobulin A may contribute to biofilm formation in the gut. Immunology. 10.1046/j.1365-2567.2003.01700.x. PMC1782994 12871226

[R30] Ren X, Akiyoshi K, Dziennis S, Vandenbark AA, Herson PS, Hurn PD, Offner H, 2011. Regulatory B cells limit CNS inflammation and neurologic deficits in murine experimental stroke. J. Neurosci 10.1523/JNEUROSCI.1623-11.2011. PMC3111929 21653859

[R31] Rojas OL, Pröbstel AK, Porfilio EA, Wang AA, Charabati M, Sun T, Lee DSW, Galicia G, Ramaglia V, Ward LA, Leung LYT, Najafi G, Khaleghi K, Garcillán B, Li A, Besla R, Naouar I, Cao EY, Chiaranunt P, Burrows K, Robinson HG, Allanach JR, Yam J, Luck H, Campbell DJ, Allman D, Brooks DG, Tomura M, Baumann R, Zamvil SS, Bar-Or A, Horwitz MS, Winer DA, Mortha A, Mackay F, Prat A, Osborne LC, Robbins C, Baranzini SE, Gommerman JL, 2019. Recirculating Intestinal IgA-Producing Cells Regulate Neuroinflammation via IL-10. Cell. 10.1016/j.cell.2018.11.035. PMC6903689 30612739

[R32] Scheeren FA, Nagasawa M, Weijer K, Cupedo T, Kirberg J, Legrand N, Spits H, 2008. T cell-independent development and induction of somatic hypermutation in human IgM + IgD + CD27 + B cells. J. Exp. Med 10.1084/jem.20070447. PMC2526198 18695003

[R33] Schuhmann MK, Langhauser F, Kraft P, Kleinschnitz C, 2017. B cells do not have a major pathophysiologic role in acute ischemic stroke in mice. J. Neuroinflammation 10.1186/s12974-017-0890-x. PMC5457733 28576128

[R34] Snoeck V, Peters IR, Cox E, 2006. The IgA system: A comparison of structure and function in different species. Res Vet. 10.1051/vetres:2006010. 16611558

[R35] Stokes J, Hoffman EA, Zeng Y, Larmonier N, Katsanis E, 2016. Post-transplant bendamustine reduces GvHD while preserving GvL in experimental haploidentical bone marrow transplantation. Br. J. Haematol 10.1111/bjh.14034. PMC4917459 27030315

[R36] Tiller T, Busse CE, Wardemann H, 2009. Cloning and expression of murine Ig genes from single B cells. J. Immunol. Methods 10.1016/j.jim.2009.08.009. 19716372

[R37] Vindegaard N, Muñoz-Briones C, El Ali HH, Kristensen LK, Rasmussen RS, Johansen FF, Hasseldam H, 2017. T-cells and macrophages peak weeks after experimental stroke: Spatial and temporal characteristics. Neuropathology. 10.1111/neup.12387. 28517732

[R38] Waldman A, Tadi P, Rawal AR, 2020. Stroke Center Certification. Treasure Island (FL). 30571013

[R39] Weitbrecht Luis, Berchtold Daniel, Zhang Tian, Jagdmann Sandra, Dames Claudia, Winek Katarzyna, Meisel Christian, Meisel Andreas, 2020. CD4+ T cells promote delayed B cell responses in the ischemic brain after experimental stroke. Brain Behav. Immun 10.1016/j.bbi.2020.09.029. 33002634

[R40] Wijburg OLC, Uren TK, Simpfendorfer K, Johansen FE, Brandtzaeg P, Strugnell RA, 2006. Innate secretory antibodies protect against natural Salmonella typhimurium infection. J. Exp. Med 10.1084/jem.20052093. PMC2118088 16390940

[R41] Zbesko JC, Nguyen TVV, Yang T, Frye JB, Hussain O, Hayes M, Chung A, Day WA, Stepanovic K, Krumberger M, Mona J, Longo FM, Doyle KP, 2018. Glial scars are permeable to the neurotoxic environment of chronic stroke infarcts. Dis Neurobiol. 10.1016/j.nbd.2018.01.007. PMC5851450 29331263

